# The upper airway microbiota: how host and environment shape this ecological niche

**DOI:** 10.1038/s41392-024-01996-w

**Published:** 2024-10-09

**Authors:** Ann-Kathrin Lederer, Kristina Endres

**Affiliations:** 1 Department of General, Visceral and Transplantation Surgery, University Medical Center Mainz, 55131 Mainz, Germany; 2 Department of Psychiatry and Psychotherapy, University Medical Center Mainz, 55131 Mainz, Germany

**Keywords:** Microbiology, Medical research

In a recent study published in *Cell*, Odendaal et al. characterized the human upper respiratory tract (URT) microbiota by using samples from 3160 Dutch individuals.^1^ They presented an atlas of the URT microbiota and were able to define associations with host and environmental factors that shape the respective ecological niches (saliva, oropharynx and nasopharynx).

**Fig. 1:**
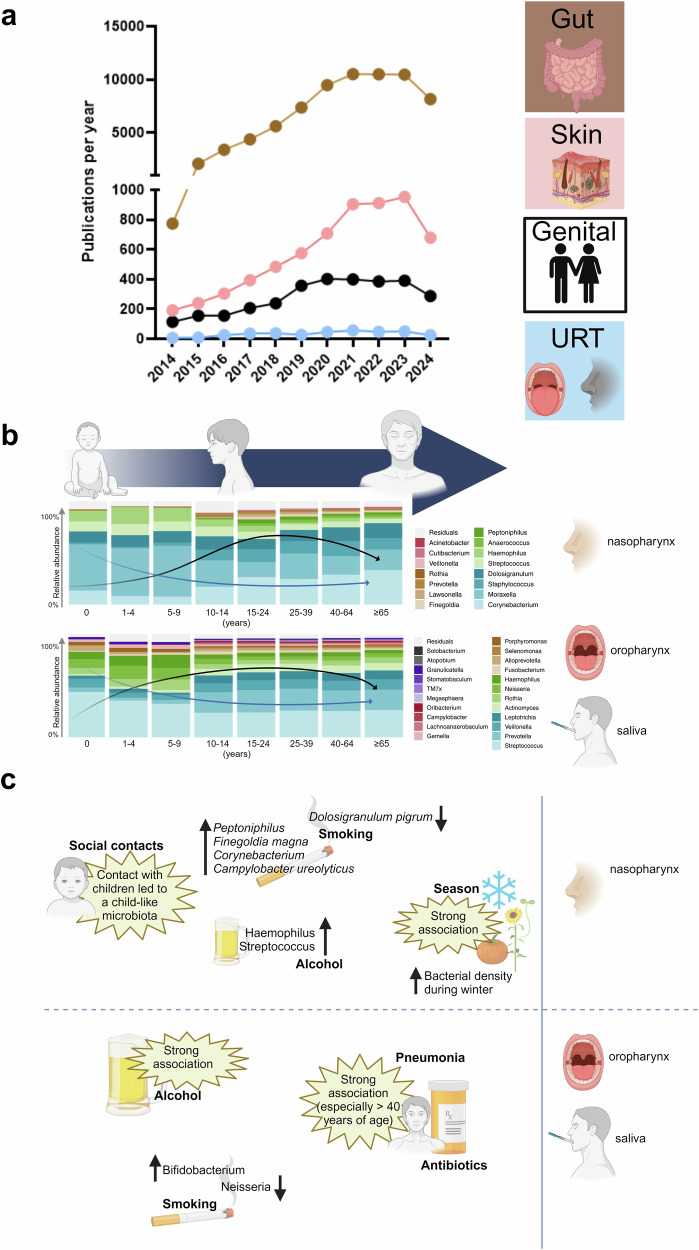
Schematic overview of publications on microbiota and key findings. **a** PubMed was searched on 14 August 2024 using the terms “x+microbiota” (x = respective body site, gut/skin/upper airway/genital). **b** Associations between URT microbiota at ASV level and different life stages adapted from Odendaal et al.^1^: the diversity increases over time (black line), while bacterial density decreases (blue line); saliva was collected from children under 10 years of age. **c** Selection of associations between URT microbiota and environmental factors observed by Odendaal et al.^1^. (Images were created with BioRender.com and GraphPadPrism8.0)

Within recent years the long-neglected microbial community that inhabits the human body has come into focus for its value as an epidemiological descriptor, but also as a potential driver of diseases that are not necessarily located in the body compartment under investigation. An example of this is Alzheimer’s disease, where researchers are still searching for early determinants of pathogenesis within the gut microbial community and oral *Porphyromonas gingivalis* has been highlighted as an airway microbe associated with this disease.^2^ Interestingly, the human body provides a wealth of defined niches for microbes, for example, each part of the skin can become its own ecosystem due to characteristics such as the moisture or fat content of the surface layer. Nevertheless, research over the last 10 years has focused mainly on the intestinal system (see Fig. 1a). Certainly, the large and small intestinal compartments have the highest number of bacteria due to their relative large volume (10^14^ for colon).^3^ However, dental plaque or saliva (bacterial counts: 10^12^ and 10^11^) and other niches have also high densities of our commensals. The URT may be of particular interest, because like the skin, it is highly exposed to environmental factors and is easily accessible for sampling. An in-depth analysis of the microbiota of the URT has therefore been the elephant in the room and this recent publication fills a gap by providing detailed insights – especially in relation to human lifespan.

Odendaal et al. found a strong separation between nasopharyngeal and oral (oropharyngeal or salivary) microbiota (see Fig. 1b).^1^ However, the niche samples showed intra-individual rather than inter-individual concordance, which has also been reported in gut microbiota research.^4^ A particularly high degree of concordance was found in infants, indicating a lack of niche differentiation at this stage. This observation is followed by another important finding, as Odendaal et al. conclude that age is the most important factor for the composition of the microbial community. Similar to the gut microbiota, the URT microbiota appears to evolve throughout life in all niches examined (see Fig. 1b).^5^

Children under 10 years of age had lower nasopharyngeal α-diversity than adolescents and adults, whereas diversity decreased again in the elderly. In contrast to diversity, the nasopharyngeal bacterial density decreased during adolescence. Different amplicon sequence variants (ASVs) were found orally (saliva and oropharynx), but the change in diversity and bacterial density was very similar to the nasopharyngeal results. The authors also identified clusters that could be assigned to the different age groups. The nasopharynx and the saliva of younger children were dominated by the *Moraxella* and *Haemophilus* clusters and the *Streptococcus* cluster, respectively.

As it is well known from other microbiota studies that origin and several influencing factors affect the gut microbiota, a clear strength of the study is that subjects were recruited from different regions of the Netherlands and that possible influencing factors were carefully considered. Odendaal et al. associated several factors such as sex, social contacts, diet, tobacco and alcohol use, antibiotic use, season of the year as well as respiratory symptoms and the occurrence of pneumonia with the URT microbiota. Unsurprisingly, but demonstrating the quality of the study, it was found that season and onset of respiratory tract infection symptoms were associated with the nasopharyngeal microbial composition. This observation mainly concerned infants and their parents, but contact with children generally resulted in a childlike microbiota with lower diversity, higher bacterial density and higher absolute abundance of *Moraxella* and *Dolosigranulum pigrum*. Interestingly, the oropharyngeal microbiota was less influenced by social contacts. Tobacco and alcohol use had different effects on the microbial composition. Tobacco use led to significant changes in the nasopharyngeal microbiota (see Fig. 1c), decreased the oropharyngeal abundance of *Neisseria* and increased the oropharyngeal abundance of *Bifidobacterium*. Alcohol had a particular strong effect on the oropharyngeal microbiota composition. In the nasopharynx, alcohol consumption led to an increased abundance of *Haemophilus* and *Streptococccus*. Antibiotic use was associated with lower microbial diversity and compositional changes in both the nasopharynx and oropharynx, with a particularly strong association in the oropharynx. A closer look at the data from Odendaal et al. reveals further influencing factors such as diet and sex, which will not be explained further due to the brevity of this text.

Overall, the publication by Odendaal et al. is a carefully prepared scientific innovation that is well worth reading.^1^ Of course, the results of microbiota studies are always limited by the diversity of the participants, but Odendaal et al. address this problem with a large sample size and the consideration of confounding factors. The authors note that they could have taken into account the subjects’ dental hygiene, which may be a relevant factor and should be considered in future URT microbiota studies. It should also be borne in mind that the results only apply to Dutch citizens and are therefore difficult to extrapolate to people from other regions of the world.
